# Novel Delivery of Cyclic-Diguanylate Monophosphate Utilizing Amyloid Depots

**DOI:** 10.3390/pharmaceutics17050668

**Published:** 2025-05-19

**Authors:** Maytham Ismail, Benjamin Beluzo, Sergei Chuikov, Venkateshwar G. Keshamouni, Mathumai Kanapathipillai

**Affiliations:** 1 Department of Mechanical Engineering, University of Michigan-Dearborn, Dearborn, MI 48128, USA; mgismail@umich.edu (M.I.); bbeluzo@umich.edu (B.B.); 2 Division of Pulmonary and Critical Care Medicine, Department of Internal Medicine, University of Michigan, Ann Arbor, MI 48109, USA; schuikov@med.umich.edu (S.C.); vkeshamo@med.umich.edu (V.G.K.); 3 LTC Charles S. Kettles VA Medical Center, Research Service (151), Ann Arbor, MI 48109, USA

**Keywords:** amyloid, RIP3, lysozyme, drug delivery, immune response

## Abstract

**Background:** Recently, cyclic diguanylate monophosphate (c-di-GMP) drug delivery has garnered interest due to its potential in cancer immune modulation. In this pilot study, we developed a novel c-di-GMP formulation based on peptide amyloids. The amyloid depots were formed by combining an amyloidogenic prone 12 amino acid peptide sequence of receptor-interacting protein kinase 3 (RIP3) with cationic lipid ALC-0315, or using lysozyme proteins. Both RIP3 and lysozyme proteins have intrinsic physiological functions. This is the first time intrinsic peptides/protein-based amyloids have been explored for c-di-GMP delivery. The main goal was to evaluate how these amyloid depots could enhance c-di-GMP drug delivery and modulate responses in RAW 264.7 macrophage-like cells. **Methods:** Physicochemical characterization and cellular assays were utilized to characterize the amyloid structures and assess the efficacy. **Results:** Our results show that amyloid aggregates significantly improve the therapeutic efficacy of c-di-GMP. When RAW 264.7 cells were treated with c-di-GMP amyloids, we observed at least a 1.5-fold change in IL-6 expression, nitric oxide (NO) production, and reactive oxygen species (ROS) production compared to treatment with 5x free c-di-GMP treatment, which suggests that this system holds promise for enhanced therapeutic effects. **Conclusions:** Overall, these findings emphasize the potential of amyloid-based delivery systems as a promising approach for c-di-GMP delivery, warranting further investigations into their potential in therapeutic applications.

## 1. Introduction

Cyclic diguanylate monophosphate (c-di-GMP) is a bacterial second messenger that serves as a potent immunomodulator by activating innate immune pathways, particularly in monocytes, granulocytes, and macrophages [1,2,3,4]. C-di-GMP activates the innate immune response via the Stimulator of Interferon Genes (STING) pathway due to its structural similarity to cGAMP, an endogenous STING agonist [2,4,5,6]. By directly binding to STING, c-di-GMP triggers a signaling cascade that leads to type I interferon production and the release of immune-activating cytokines. C-di-GMP has been used as a model immune stimulant in several systems, including as a vaccine adjuvant [2], and for the prevention of bacterial growth [3]. Although c-di-GMP represents a promising new class of immunotherapies, effective intracellular delivery remains a major challenge due to its hydrophilic nature, which limits cell membrane transport, and susceptibility to enzymatic degradation; therefore delivery systems are required for transport [1,2,7]. Peptide nanotubes and silica nanoparticles have been explored before [8,9]. This study explores and compares two distinct delivery systems for c-di-GMP: lysozyme amyloid-based depots and RIP3 peptide/cationic lipid amyloids. 

Protein and peptide-based amyloid aggregation delivery systems have garnered increasing attention for enhancing therapeutic efficacy, improving drug stability particularly for immunomodulation and inflammation regulation [10,11,12,13]. Amyloidogenic proteins, known for their spontaneous self-assembly into nano-scale structures, have emerged as potential drug carriers for clinical applications [14]. While amyloid fibrils are often associated with pathological conditions including inflammation and disease progression, beneficial amyloid aggregates also play important physiological roles and have been successfully utilized in various biomedical applications [11,12,13]. By leveraging these properties, amyloid-based drug delivery platforms offer unique advantages over conventional systems by combining structural stability with intrinsic therapeutic effects [15,16,17,18].

Lysozyme is a model protein commonly used in amyloid-based drug delivery due to its well-documented amyloidogenic properties and structural stability [19,20]. Moreover, its cationic nature [21,22] makes it suitable for delivering nucleotides. It has been shown to exhibit strong binding affinities for various oligonucleotides, DNA, and RNA [20,23,24,25]. Additionally, lysozyme is highly expressed by hematopoietic cells and plays an important role in the defense and activation of the innate immune system [20,26]. Given lysozyme’s properties, amyloid structure, and binding affinity for oligonucleotides, it offers a promising method for delivering c-di-GMP, enhancing therapeutic efficacy in immune responses. In addition, amyloid aggregates based on the receptor-interacting protein kinase 3 (RIP3) peptide have demonstrated potential as efficient delivery depots, showing enhanced therapeutic effects [27]. Additionally, RIP3’s amyloidogenic properties and role in the activation of necroptosis and inflammatory response, with its ability to interact with hydrophobic molecules [28,29,30], may provide an effective platform to interact with the cationic hydrophobic lipid ALC-0315, which is widely used in nucleotide delivery [31,32,33], and to spontaneously form cationic amyloids for nucleotide delivery. However, the potential of combining ALC-0315 with protein/peptide-based amyloid for therapeutic drug delivery of nucleotides and immunostimulant therapies remains unexplored. Hence, this could provide another promising method for the delivery of c-di-GMP with enhanced therapeutic effects, given the properties of both ALC-0315 and RIP3 in drug depot applications.

This study investigates the potential of lysozyme-based amyloid aggregates and RIP3/ALC-0315 amyloids for delivering c-di-GMP. Physicochemical characterization and cellular efficacy studies were conducted. By enhancing stability and bioavailability, these strategies could improve c-di-GMP’s therapeutic applications.

## 2. Materials and Methods

### 2.1. Materials

Receptor-interacting protein RIP 3 peptide NIYNCSGVQVGD was custom-synthesized by Genscript (Piscataway, NJ, USA). RAW 264.7 cell lines were cultured in the laboratory and used for the study. The cell culture reagents—DMEM (Dulbecco’s Modified Eagle Medium), Antibiotic-Antimycotic, Fetal Bovine Serum, Lysozyme (12650-88-3), and Texas Red (T6134)—were obtained from Thermo Fisher Scientific (Waltham, MA, USA). DMSO (276855), Ammonium acetate (A1542), Thioflavin-T (ThT) (T3516), and Phosphotungstic acid (HT152) were obtained from Millipore Sigma (Milwaukee, WI, USA). The Alamar blue cell viability reagent (DAL 1025) and Mouse IL-6 ELISA Kit (KMC0061) were obtained from Invitrogen (Waltham, MA, USA). The Griess reagent (ALX-400004) was obtained from Enzo Life Sciences (Farmingdale, NY, USA). Cyclic di-GMP (17144) and ALC-0315 (34337) were obtained from Cayman Chemical (Ann Arbor, MI, USA). The Cyclic-di-GMP assay (SKU: 200-100) was obtained from Lucerna (Brooklyn, NY, USA). MitoROS 580 (16052) was obtained from AAT Bioquest (Pleasanton, CA, USA).

### 2.2. Cell Culture

RAW 264.7 macrophage cells, commonly used in biological and immunological research, were obtained from the American Type Culture Collection (ATCC; Manassas, VA, USA) and expanded in Dulbecco’s Modified Eagle Medium (DMEM) supplemented with 10% fetal bovine serum (FBS) and 1% antibiotic antimycotic in a humidified incubator at 37°C with 5% CO_2_. RAW 264.7 cells were cultured in 96-well or 24-well plates at densities of 10,000 cells/well and 50,000 cells/well, respectively.

### 2.3. Development of Receptor-Interacting Protein Peptide and Cyclic-di-GMP Amyloid Formulation

The peptide sequence of NIYNCSGVQVGD (RIP3) was used in this study. Initially, we prepared a 50 mM stock solution of the peptide by dissolving it in dimethyl sulfoxide (DMSO). Following this, we prepared a 2.5 mM concentration of the peptide by diluting it in 100 mM Ammonium Acetate at pH 7 to a final concentration of 1.125 mM and adjusted to pH2. Next, we added 2 µg of ALC-0315 to the peptide solution (at a molar ratio of 1:43) to facilitate the formation of RIP3/ALC-0315 (RIP3-A) amyloid depots. The mixture was then incubated at 37°C in the thermomixer (shaking incubator) at 300 rpm for 24 h to allow enough time for peptide aggregation. After 24 h, 100 µM of c-di-GMP (11:1 RIP3:c-di GMP molar ratio) was added to the aggregated mixture, and the mixture was allowed to react for an additional hour. Following this, dialysis was performed using a 3500 molecular weight cut-off (MWCO) membrane for 24 h to remove any unbound molecules to obtain the final RIP3-A-c-di-GMP formulation. Experiments were performed in triplicate to ensure consistent reproducibility. 

### 2.4. Development of Lysozyme and Cyclic-di-GMP Amyloid Formulation

The aggregation of lysozyme was performed with modifications from previously reported study [34]: 5 mg/mL of lysozyme was dissolved in 150 mM sodium chloride at pH 2. The aggregates were incubated at approximately 60°C in a thermomixer at 300 rpm for 24 h to facilitate protein aggregation. After 24 h, 100 µM of c-di-GMP (17:1 lysozyme:c-di-GMP molar ratio) was added to the aggregated mixture and the solution was allowed to react for an hour to promote electrostatic interaction between the positively charged lysozyme protein and negatively charged c-di-GMP. The one-hour aggregation was chosen to minimize c-di-GMP exposure to low pH and high temperature. Dialysis was performed to purify the aggregation using 3500 MWCO membrane obtained from Thermo Fisher Scientific (Rockford, IL, USA) for 24 h to remove any unbound molecules. After dialysis, the aggregates with c-di-GMP were obtained (Lyz-c-di-GMP). The amount of c-di-GMP in both the formulations was characterized using the c-di-GMP assay. For uptake studies, peptides and/or lysozyme were labeled with Texas Red, and similar procedures were used to produce the amyloids. Experiments were independently repeated three times.

### 2.5. Size, Surface Charge, and Morphology

Dynamic Light-Scattering (DLS) measurements were used to determine the size of the RIP3-A, both with and without c-di-GMP, as well as Lysozyme, with and without c-di-GMP. First, the samples were diluted eleven times. Water was used to dilute the samples to reflect post-dialysis conditions. Further, the stability of the aggregates under these conditions were confirmed by TEM and ThT assays. A total of 50 µL of distilled water was mixed with 5 µL of the formulation sample after dialysis in a cuvette, and size measurements were carried out using a Malvern zeta sizer instrument. Zeta potential was also measured using the same instrument to evaluate the surface charge of the formulations. For this, 70 µL of the samples was mixed with 700 µL of distilled water, loaded into disposable folded capillary cells, and measured at 25°C. Dynamic light scattering and zeta potential measurements were performed in triplicate for each formulation.

Transmission Electron Microscopy (TEM) imaging was used to assess the morphology of the samples. For TEM analysis, formulations were placed on a 400-mesh copper grid and 2% phosphotungstic acid at pH 7.4 were applied as a contrasting agent. Imaging was performed using a JEOL TEM microscope at the University of Michigan Ann Arbor Medical School’s Electron Microscopy Facility.

### 2.6. Thioflavin T (ThT) Fluorescence

Aggregation was confirmed through Thioflavin T (ThT) Fluorescence measurements. To perform the assay, a total of 100 µL of 50 µM of ThT in 20 mM of Trizma hydrochloride (Tris) buffer at pH 8 was mixed with 5 µL of the aggregation sample (20 x dilution), and the aggregation formation was assessed after dialysis at 48 h at 440 nm excitation and emission at 482 nm. The fluorescence of the solution was measured using a SpectraMax M3 spectrophotometer from Molecular Devices (San Jose, CA, USA). All ThT assays were conducted in triplicate. Fluorescence values were compared using unpaired Student’s t-tests to assess differences in aggregation levels.

### 2.7. Cyclic-di-GMP Calibration and Encapsulation

The c-di-GMP assay was employed to determine the concentration of c-di-GMP in the formulation. The assay was performed using the working reagents provided in the Lucerna c-di-GMP kit. First, standard working stocks were prepared to derive the standard calibration curve, which is essential for accurately estimating the c-di-GMP concentration in the formulation. A concentration range of 0–125 nM of the c-di-GMP was utilized to construct this curve. Fluorescence measurements were performed with an excitation wavelength of 482 nm and an emission wavelength of 505 nm to detect the presence and quantify the concentration of c-di-GMP. Next, the c-di-GMP concentration in the RIP3-A formulation and Lysozyme formulation was measured by comparing the fluorescence data to the standard calibration curve, which was based on the linear equation (y = 0.5301x + 22.22). To assess encapsulation efficiency, the initial concentration of 100 µM c-di-GMP in the formulation was compared to the amount remaining after dialysis. The ratio of the remaining c-di-GMP to the initial amount provided a measure of the encapsulation efficiency. Fluorescence measurements and encapsulation efficiency calculations were based on three independent assays per formulation. Standard curves were constructed using known c-di-GMP concentrations, and linear regression analysis was applied.

### 2.8. Cellular Uptake

To assess the cell uptake of the formulation, RAW 264.7 macrophages, a widely used immune cell line, were employed in the study. The cells were cultured in a 24-well plate at a density of 50,000/well for 24 h. The next day, the cells were treated with RIP3-A-c-di-GMP formulation and Lyz-c-di-GMP formulation, both labeled with Texas Red for 30 min. After treatment in Dulbecco’s Modified Eagle Medium (DMEM) cell culture media, the cells were washed several times and then resuspended in PBS buffer. The cell suspension was placed on ice to preserve the samples. Cellular uptake was then analyzed using the Attune NxT flow cytometry from Thermo Fisher Scientific (Waltham, MA, USA) available in the lab. For the study, a 488 nm blue laser (excitation/emission 488/590 nm) was used. Amyloid uptake was considered significant for Texas red value above 10^4^. Cells without any treatment were used as negative control. Each treatment included three replicates. Cells without any treatment were used as controls.

### 2.9. Cellular Toxicity

RAW 264.7 macrophages were employed for the cellular toxicity study. Cells were cultured in Dulbecco’s Modified Eagle Medium (DMEM) supplemented with 10% fetal bovine serum (FBS) and 1% antibiotic-antimycotic solution to maintain optimal growth conditions. The cells were cultured at a density of 10,000 cells/well in a 96-well plate. After 24 h of incubation, the cells were treated with 1, 5, 10 µM of c-di-GMP alone, ALC-0315 (A) alone, 1 µM of RIP3-A-c-di-GMP formulation, and 1 µM Lyz-c-di-GMP formulation. The amount of c-di-GMP was determined by c-di-GMP assay. Following the 48 h of incubation, metabolic activity was assessed using Alamar blue assay. Cell toxicity was assessed according to the metabolic activity of the cells at excitation of 570 nm and emission at 590 nm. Each treatment condition was tested in triplicate and repeated across three independent experiments. Untreated cells were used as controls.

### 2.10. Nitric Oxide (NO) Assay and Mitochondrial ROS (MitoROS)

RAW 264.7 macrophages were used to assess the potential immune response induced by protein aggregates, and the nitric oxide (NO) and Mitochondrial ROS (MitoROS) assays were conducted. The Griess reagent assay and the MitoROS assay were performed. The cells were cultured at 10,000 cells/well in a 96-well plate. After 24 h incubation, the cells were treated with 1, 5, 10 µM of c-di-GMP alone, ALC-0315 (A) alone, 1 µM of RIP3-A-c-di-GMP formulation, and 1 µM Lyz-c-di-GMP formulation. Following another 24 h of incubation, the production of NO in the culture media was measured using equal volume of Griess reagent in the dark at room temperature for 15 min. This assay detects NO by measuring the concentration of nitrite, which was determined by measuring absorbance at 540 nm. Additionally, 48 h after adding the formulation to the cell, mitochondrial ROS was assessed using the AAT Bioquest MitoROS 580 assay (Pleasanton, CA, USA). First, MitoROS 580 stock solution was made by dissolving MitoROS 580 in 13 µL of DMSO, then MitoROS 580 working solution was created by diluting stock solution 500X with sterile PBS buffer. Next, cells were treated with equal volumes of MitoROS 580 working solution for 30 min. Cells were then washed three times with sterile PBS buffer. Fluorescence intensity was measured at excitation and emission wavelengths of 500 nm and 582 nm, respectively. All conditions were tested in triplicate and the assays were independently replicated at least three times. Controls included untreated cells.

### 2.11. IL-6 ELISA Assay

IL-6 ELISA assay was performed to evaluate whether the formulation induces an immune response. Raw 264.7 macrophage cells were cultured in 96-well plate at density of 10,000 cells/well. After 24 h of incubation, the cells were treated with 1, 5 µM of c-di-GMP alone, 1 µM of RIP3-A-c-di-GMP formulation, and 1 µM Lyz-c-di-GMP formulation. After 24 h, the immune response was assessed by measuring the level of IL-6 in the culture supernatants. IL-6 concentrations were quantified using the Mouse IL-6 ELISA Kit, from Invitrogen (Waltham, MA, USA), following the manufacturer’s protocol, and absorbance measured at 450 nm. IL-6 concentrations were measured in triplicate wells for each treatment group and compared using one-way ANOVA. Cells treated with only media served as controls.

### 2.12. Statistical Analysis

All experiments in this study were conducted with a minimum of three independent repetitions to ensure reliable results. Data are presented as the mean ± standard error of the mean (SEM). The analysis was carried out using Prism software (version 9.20) and Microsoft Excel (version 16.69). Statistical significance was assessed using analysis of variance (ANOVA) followed by unpaired student t-tests or Tukey’s HSD post hoc tests, depending on the experimental design. The following significance levels were used: * *p <* 0.05, or ** *p <* 0.01, *** *p <* 0.001, **** *p <* 0.0001.

## 3. Results

### 3.1. Characterization and Efficacy Studies

#### 3.1.1. Physicochemical Characterization

**Figure 1:**
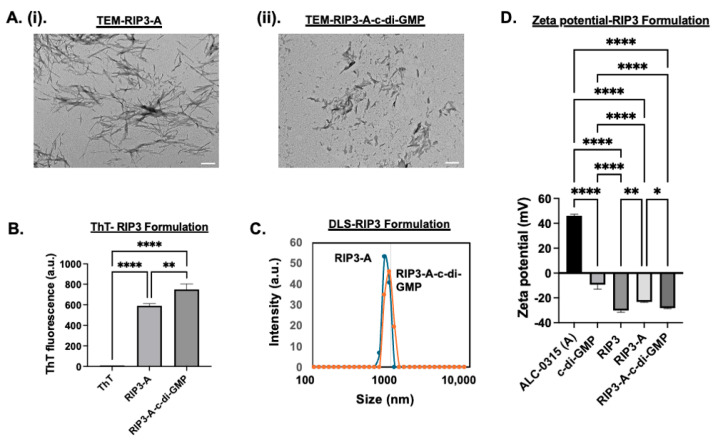
Characterization of RIP3-A aggregation in the presence and absence of c-di-GMP. (**A**) TEM images. Scale bar: 200 nm. (**i**) TEM of RIP3-A without c-di-GMP. (**ii**) TEM of RIP3-A with c-di-GMP. (**B**) ThT fluorescence measurements confirming amyloid-like aggregation of RIP3 formulation. (**C**) DLS size distribution of RIP3 formulation. (**D**) Zeta potential measurements. Three independent experiments (n=3) were conducted. * *p <* 0.05, ** *p <* 0.01, **** *p <* 0.0001.

First, amyloid formulations were characterized for RIP3/ALC-0315 (RIP3-A) in the presence and absence of c-di-GMP. TEM images and DLS measurements were carried out to determine size and morphology, while zeta potential was analyzed for charge and aggregation behavior, and ThT measurements at 440/482 nm excitation/emission were performed to confirm amyloid formation (Figure 1A–D). The rationale of the combo system was based on zeta potential to analyze surface charge and electrostatic interactions. RIP3 had a zeta potential of −30.2 ± 0.85 mV, and c-di-GMP had a zeta potential of −9.5 ± 2.1 mV. Given the anionic nature of both RIP3 and c-di-GMP, incorporation of c-di-GMP would not occur because of charge repulsion; hence, the introduction of a cationic particle was needed. ALC-0315 was measured to have a zeta potential of 46 ± 0.72 mV. The combo RIP3-A had a zeta potential of −23.27 ± 0.25 mV, and RIP3-A with c-di-GMP had a decreased zeta potential of −28.27 ± 0.27 mV, likely indicating incorporation of c-di-GMP. TEM analysis confirmed the amyloid-like morphology in all conditions. RIP3-A alone amyloids revealed longer and thinner fibrillar structures, whereas RIP3-A with c-di-GMP showed shorter and thicker fibrillar structures but demonstrated greater clumping compared to RIP3-A alone, as shown in Figure 1A(i,ii). RIP3-A exhibited significant aggregation, observed both in the presence and absence of c-di-GMP (*p*-value < 0.0001), as shown in Figure 1B. RIP3-A with c-di-GMP had significantly enhanced aggregation compared to RIP3-A alone (*p*-value < 0.01), as depicted by ThT fluorescence in Figure 1B; however, this result might be due to clumping of the aggregates rather than increased aggregation. DLS measurements demonstrated a rightward shift in size distribution upon c-di-GMP incorporation as shown in Figure 1C. The average size of RIP3-A alone was 897.19 ± 12.02 nm, and that of RIP3-A with c-di-GMP was 1020.49 ± 18.61 nm; this, along with a zeta potential decrease, suggested a successful incorporation of c-di-GMP into the RIP3-A amyloid aggregate. This observation is in agreement with ThT fluorescence, which indicated enhanced aggregation and structural differences between RIP3-A with and without c-di-GMP, as shown in Figure 1A–C.

**Figure 2:**
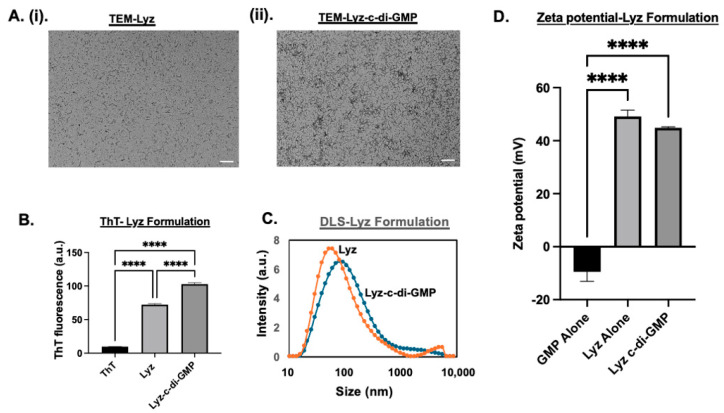
Characterization of lysozyme aggregation in presence and absence of c-di-GMP. (**A**) TEM images. Scale bar: 200 nm. (**i**) TEM of lysozyme without c-di-GMP. (**ii**) TEM of lysozyme with c-di-GMP. (**B**) ThT fluorescence measurements confirm significant aggregation of lysozyme. Aggregation is significantly enhanced by c-di-GMP. (**C**) DLS size distribution of lysozyme formulation. (**D**) Zeta potential measurements. Three independent experiments (n = 3) were performed. **** *p <* 0.0001.

Next, lysozyme amyloid formulations (Lyz) were characterized. The zeta potential of Lyz alone was 49.3 ± 1.42 mV. The strong cationic nature of Lyz demonstrates that it does not require the incorporation of ALC-0315 to interact with c-di-GMP. Lysozyme-only aggregates showed very short, low-density fibrillar structures, whereas Lyz with c-di-GMP formed structures were denser and had longer fibrillar structures, as shown in Figure 2A(i,ii). A similar trend was also observed in lysozyme aggregates with and without c-di-GMP, indicating significant aggregation (*p*-value < 0.0001), as shown in Figure 2B. Additionally, there was a significant increase in aggregation for Lyz with c-di-GMP compared to Lyz alone (*p*-value < 0.0001) Figure 2B. In comparison, RIP3-A aggregates exhibited higher ThT fluorescence compared to lysozyme aggregates shown in Figure 1B and Figure 2B. The average size of Lyz alone was found to be 63.55 ± 1.86 nm, and Lyz with c-di-GMP was found to be 84.263 ± 4.05 nm. A decrease in zeta potential was also observed when c-di-GMP was incorporated, dropping to 44.86 ± 0.21 mV, indicating likely incorporation of c-di-GMP. This increase in hydrodynamic diameter suggests the incorporation of c-di-GMP into the lysozyme aggregates and is further confirmed by ThT fluorescence, zeta potential, and TEM, which all indicated increased size, density, aggregation, and differences in morphology, as shown in Figure 2A–D. Furthermore, DLS measurements and TEM provide an explanation for why there is an increased ThT fluorescence of RIP3-A compared to Lyz, as RIP3-A demonstrates longer and thicker fibrillar aggregates compared to lysozyme.

#### 3.1.2. Cellular Efficacy Studies

**Figure 3:**
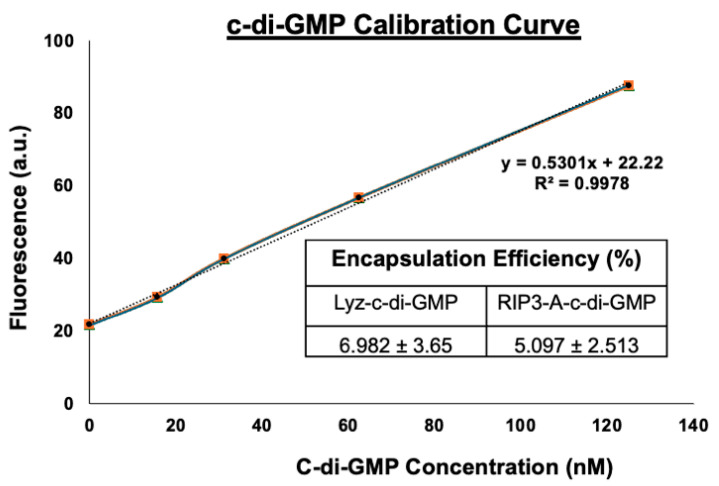
Quantification of c-di-GMP encapsulation in RIP3-A and lysozyme formulation. The c-di-GMP encapsulation was measured using Lucerna Cyclic-di-GMP assay with a calibration curve for c-di-GMP concentrations ranging from 0 to 125 nM. Three independent experiments were used to determine the encapsulation efficiency for both RIP3-A and lysozyme.

**Figure 4:**
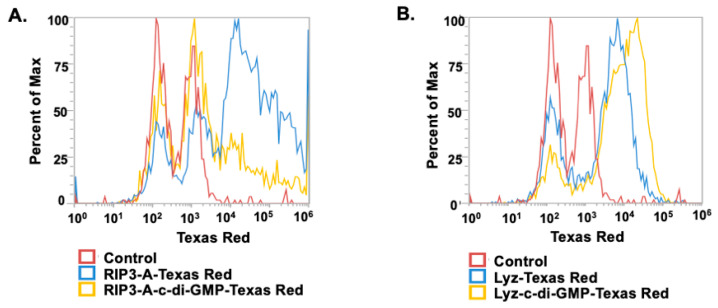
Cellular uptake of RIP3-A and lysozyme formulation with and without c-di-GMP in RAW 264.7 macrophage cells using flow cytometry. RAW 264.7 macrophage cells were cultured in a 24-well plate and treated with RIP3-A-c-di-GMP and Lyz-c-di-GMP formulations, both were labeled with Texas Red for 30 min, and then uptake was analyzed. Here, the y-axis represents the percentage of detected fluorescence compared to that of the maximum detected fluorescence value: (**A**) the RIP3-A formulation showed a greater fluorescence shift compared to the control; (**B**) the lysozyme formulation exhibited a rightward shift in fluorescence intensity compared to the control.

**Figure 5:**
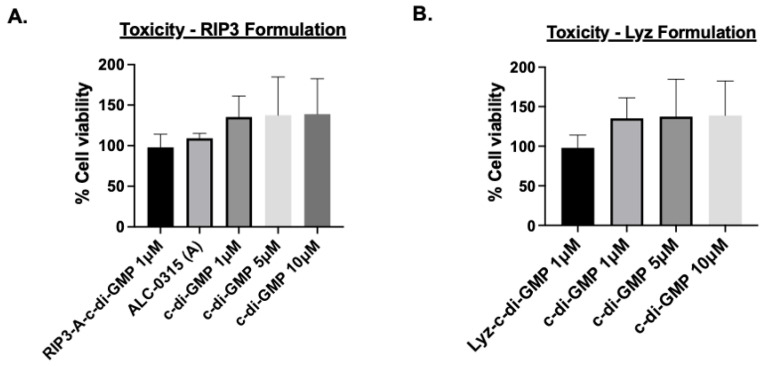
Cytotoxicity assessment of RIP3-A and lysozyme formulation (Lyz) with c-di-GMP in RAW 264.7 macrophage cells using the Alamar blue assay. RAW 264.7 cells were cultured in a 96-well plate and treated with 1, 5, 10 µM of c-di-GMP alone, (**A**) 1 µM of RIP3-A-c-di-GMP formulation, and (**B**) 1 µM Lyz-c-di-GMP formulation. Neither RIP3 nor lysozyme formulations caused significant toxicity compared to the control. Similarly, all tested concentrations of c-di-GMP did not show any toxic effects. Three independent experiments (n = 3) were performed.

Given the amyloid structures observed for both RIP3-A and Lyz, further investigation into cellular efficacy was warranted for potential depots of oligonucleotides. To quantify c-di-GMP encapsulation, a Lucerna Cyclic-di-GMP assay was employed to generate a calibration curve for c-di-GMP concentrations ranging from 0 to 125 nM. The amount of c-di-GMP encapsulated for both samples was determined from the equation (y = 0.5301x + 22.22 mol/mol), as shown in Figure 3. The average encapsulation for RIP3-A was found to be 5.097 ± 2.513%. The average encapsulation for Lyz was found to be 6.982 ± 3.65%. Subsequently, flow cytometry was used to assess cellular uptake of RIP3-A and Lyz, both with and without c-di-GMP, in RAW 264.7 macrophage cells derived from murine leukemia tumors. As shown in Figure 4A,B, all Texas Red labeled aggregate treated samples exhibited a rightward shift in Texas Red fluorescence intensity compared to untreated cells, indicating successful cellular uptake. Comparing RIP3-A with and without c-di-GMP, there was a greater rightward shift for RIP3-A alone compared to that with c-di-GMP, indicating increased cellular uptake. This increase in uptake for RIP3-A alone might be due to a lower overall negative charge compared to RIP3-A with c-di-GMP, as revealed by zeta potential measurements. On the other hand, Lyz with c-di-GMP has a greater rightward shift than Lyz alone. Although no significant difference in zeta potential was observed, this may be due to significant aggregation indicating an increased cellular uptake of Lyz-c-di-GMP. Next, we investigated if there were potential cytotoxic effects of RIP3-A and lysozyme aggregates with c-di-GMP. RAW 264.7 macrophage cells were used for toxicity and subsequent cellular studies. An Alamar blue assay was conducted, where cells were treated with aggregate samples containing 1 µM c-di-GMP, as well as free c-di-GMP at concentrations of 1 µM, 5 µM, and 10 µM, based on previously established efficacious concentrations [35,36]. As shown in Figure 5A,B, neither RIP3-A nor Lyz aggregates with c-di-GMP exhibited any significant toxicity compared to controls. Similarly, all tested concentrations of free c-di-GMP showed no toxic effects, confirming its biocompatibility under these conditions. The % cell viability was calculated based on the viability of untreated cells.

**Figure 6:**
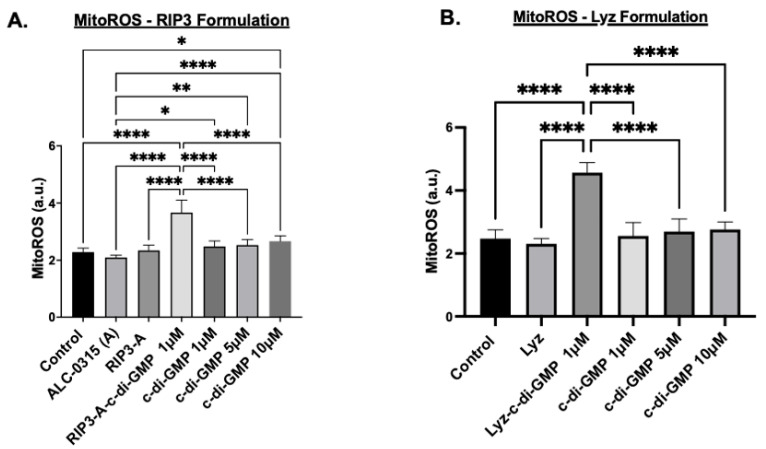
Immune response activation of RIP3-A and lysozyme formulation (Lyz) with and without c-di-GMP in RAW 264.7 macrophages. RAW 264.7 macrophage cells were cultured in a 96-well plate and treated with 1 µM, 5 µM, 10 µM of c-di-GMP alone, (**A**) 1 µM of RIP3-A formulation with and without c-di-GMP, and (**B**) 1 µM Lyz formulation with and without c-di-GMP. Both RIP3-A and lysozyme formulation with c-di-GMP exhibited significant increase in MitoROS production compared to untreated cells, formulation without c-di-GMP, and c-di-GMP at concentrations of 1 µM, 5 µM, 10 µM. Three independent experiments (n = 3) were used. * *p <* 0.05, ** *p <* 0.01, **** *p <* 0.0001.

Further tests were carried out to assess the potential of RIP3-A and lysozyme aggregates as drug depots for oligonucleotide delivery and inflammatory response induction; multiple assays were conducted to confirm immune activation. The first study measured mitochondrial reactive oxygen species (MitoROS) production in the cells treated with RIP3-A and Lyz with and without c-di-GMP, as well as free c-di-GMP. The concentrations of all samples matched those used in the cytotoxicity studies, and aggregates without c-di-GMP prepared in volumes equivalent to their c-di-GMP-containing counterparts. Cells were treated with MitoROS 580 working reagent from the AAT Bioquest MitoROS assay, and fluorescence was measured at 500/582 nm excitation/emission. The results for RIP3-A aggregates showed that RIP3-A with c-di-GMP exhibited a significant increase in MitoROS production compared to all other conditions, including the untreated control, RIP3-A alone, and free c-di-GMP at 1 µM, 5 µM, and 10 µM (*p*-value < 0.0001), as shown in Figure 6A. A similar trend was observed for lysozyme aggregates, where Lyz with c-di-GMP induced a significant increase in MitoROS production compared to Lyz alone and free c-di-GMP at 1 µM, 5 µM, and 10 µM (*p*-value < 0.0001) Figure 6B. Among the free c-di-GMP conditions, only the highest concentration (10 µM) resulted in a significant increase in MitoROS production relative to the control (Figure 6A). 

**Figure 7:**
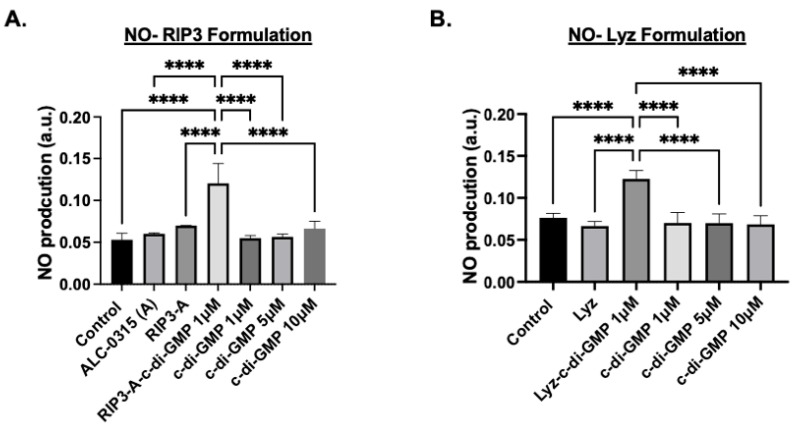
Nitric oxide (NO) production using the Griess reagent assay in RAW 264.7 cells. RAW 264.7 macrophage cells were cultured in a 96-well plate and treated with 1 µM, 5 µM, 10 µM of c-di-GMP alone, (**A**) 1 µM of RIP3-A formulation with and without c-di-GMP, and (**B**) 1 µM Lysozyme formulation with and without c-di-GMP. NO production was measured the absorbance at 540 nm. Both RIP3-A and lysozyme formulation with c-di-GMP showed a significant increase in NO production compared to control. Three independent experiments (n = 3) were employed. **** *p <* 0.0001.

Next, nitric oxide (NO) production measurements were used as an indicator of the inflammatory response since macrophages produce NO as a signaling molecule and are an indication of the activation of immune cells [37]. The same experimental conditions used in the MitoROS assay were applied, and NO production was quantified using the Griess reagent assay with absorbance measured at 540 nm. The results for RIP3-A aggregates showed that RIP3-A with c-di-GMP indicated a significant increase in NO production compared to all other conditions, including control, RIP3-A alone, and all free c-di-GMP concentrations (*p*-value < 0.0001), as shown in Figure 7A. Similarly, Lyz with c-di-GMP indicated the same trend as RIP3-A with c-di-GMP, indicating a significant increase in NO production compared to control, lysozyme alone, and all free c-di-GMP concentrations (*p*-value < 0.0001) (Figure 7B). 

**Figure 8:**
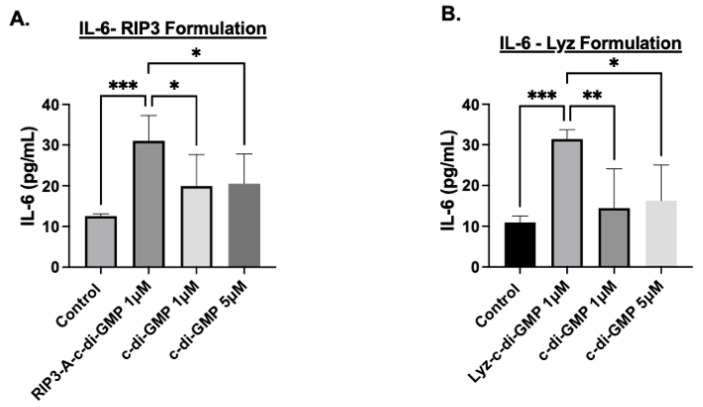
IL-6 cytokine production using ELISA to assess the inflammatory response in RAW 264.7 cells. RAW 264.7 macrophage cells were cultured in a 96-well plate and treated with 1 µM and 5 µM of c-di-GMP alone, and (**A**) 1 µM of RIP3-A formulation with c-di-GMP. RIP3-A-c-di-GMP exhibited significantly higher IL-6 expression compared to the control. (**B**) 1 µM lysozyme formulation with c-di-GMP. Similarly, lyz-c-di-GMP caused a significant increase in IL-6 expression compared to the control and the all concentrations of c-di-GMP. Three independent experiments (n = 3) were performed. * *p <* 0.05, ** *p* < 0.01, *** *p <* 0.001.

Finally, the production of the cytokine IL-6 was used to validate the inflammatory response; IL-6 cytokine expression was measured using ELISA. The results for RIP3-A with c-di-GMP induced a highly significant increase in IL-6 expression compared to control (*p*-value < 0.001) (Figure 8A). Additionally, IL-6 expression in RIP3-A with c-di-GMP was significantly higher than in cells treated with 1 µM and 5 µM free c-di-GMP (*p*-value < 0.05), though the difference was not as pronounced as observed with the control, shown in Figure 8A. Similarly, Lyz with c-di-GMP indicated the same trend as RIP3-A with c-di-GMP, indicating a highly significant increase in the expression of IL-6 compared to control (*p*-value < 0.001) (Figure 8B). Further, Lyz with c-di-GMP indicated a significant expression compared to 1µM and 5 µM c-di-GMP but not as strong as it compared to control (*p*-value < 0.05). Although the magnitude of c-di-GMP immune response is smaller compared to lipopolysaccharide (LPS), a potent immune system activator [38], collectively, the results from MitoROS production, NO production, and IL-6 cytokine expression after just 24 h of treatment strongly indicate that RIP3-A and Lyz amyloid aggregates function as effective drug depots for oligonucleotide delivery, leading to a robust inflammatory response. These findings highlight their potential for immunostimulatory applications and further validate the role of c-di-GMP in enhancing aggregate-mediated immune activation.

## 4. Discussion

This paper presents findings highlighting the potential of RIP3-A and Lyz amyloid aggregates as drug depots for oligonucleotides to enhance immune stimulation. The study reveals significant aggregation and the formation of amyloid-like structures in both RIP3-A and lysozyme, with and without c-di-GMP, as confirmed by ThT fluorescence. Notably, RIP3-A demonstrated higher fluorescence levels, both in the presence and absence of c-di-GMP, compared to the lysozyme conditions, indicating more substantial fibrillar aggregation. This observation may be linked to longer amyloid aggregates and a greater density of aggregates, as supported by previous studies [27]. Moreover, incorporating ALC- 0315 into the RIP3 aggregate structure might influence the aggregation kinetics, physicochemical properties, and electrostatic interaction given by the large surface charge difference, contributing to the enhanced fibrillar aggregation. The observed increase in aggregation with c-di-GMP incorporation in both amyloid aggregates indicates that c-di-GMP was encapsulated within the structure and that cyclic dinucleotides might be able to modulate protein aggregation dynamics. Structural analysis via transmission electron microscopy, zeta potential, and DLS measurements validated the ThT fluorescence results, showing that RIP3-A had thicker, longer, and denser fibrillar aggregates compared to lysozyme, which formed shorter and thinner fibrillar structures. The hydrodynamic diameter shift for RIP3-A from 897.19 ± 12.0 nm to 1020.49 ± 18.61 nm and for Lyz from 63.55 ± 1.86 nm to 84.263 ± 4.05 nm further supports the encapsulation and incorporation of c-di-GMP into the amyloid aggregates. It is important to note that while DLS could be used to measure the size, it may not predict the size of the amyloid structures with high accuracy due to the heterogeneity of the amyloids. These amyloid structures achieved encapsulation efficiencies of approximately 5% for RIP3-A and around 7% for lysozyme. The relatively low encapsulation efficiencies may have resulted from the brief one-hour binding time. Additionally, the purification process might have eliminated any c-di-GMP that was weakly associated with the surfaces of both aggregates, thereby reducing the overall encapsulation efficiency. The pH, concentrations, reaction time, reaction temperature, and dialysis conditions need to be optimized to enhance encapsulation efficiency. 

First, it is important, before cellular studies, to discuss the toxic nature of amyloids and other protein aggregates. Traditionally, amyloids are associated with a range of pathological conditions, including neurodegenerative diseases, diabetes, amyloidosis, liver disease, and kidney failure [39,40]. Amyloids are misfolded proteins that form insoluble structures resistant to proteolysis, leading to the accumulation of amyloids in tissues, causing the breakdown of metabolism and organ functions [40,41]. Certain amyloid-like proteins, such as prions, are even infectious; they induce misfolding in other proteins, further compromising cellular function [42]. Although a variety of amyloid structures lead to disease, there has been a recent discovery of functional amyloids in a variety of eukaryotes, mammals, and even humans [39,43,44,45]. Among such proteins that have been found as functional amyloids and safe for use are lysozyme and RIP3, their safety supported by cytotoxicity assays and other validation studies [20,27,46]. 

Cellular studies further demonstrated the potential of amyloid aggregates as depots for immune stimulation. Cytotoxicity assays confirmed the biocompatibility of RIP3-A and lysozyme aggregates, as well as free c-di-GMP, at the tested concentrations in RAW 264.7 macrophages. To evaluate their immunostimulatory potential, we assessed mitochondrial ROS production, NO production, and IL-6 expression as markers of immune activation. RIP3-A and lysozyme encapsulating c-di-GMP elicited significantly higher levels of all three markers compared to both free c-di-GMP and aggregates alone. Notably, encapsulating the lowest tested c-di-GMP concentration (1 µM) into both aggregates, they still induced a significantly greater immune response than the highest free c-di-GMP concentration (10 µM), suggesting enhanced efficacy. Both RIP3-A and lysozyme aggregates loaded with c-di-GMP induced significantly higher mitochondrial ROS and NO production than free 10 µM c-di-GMP, with comparable responses observed between the two aggregate types. This indicates that both aggregates function effectively as delivery platforms, with neither demonstrating superior immune activation. The IL-6 cytokine expression results corroborated these findings, reinforcing the conclusion that c-di-GMP-loaded aggregates significantly enhance immune responses. Collectively, these findings highlight the potential of RIP3-A and lysozyme aggregates as immunostimulatory carriers capable of enhancing innate immune responses through a c-di-GMP delivery. A more comprehensive study incorporating multiple dose levels and time points could provide valuable data to aid in the development of optimized formulations.

In summary, this study demonstrates the potential of RIP3-A and lysozyme-based amyloid aggregates as drug depots for the delivery of c-di-GMP to enhance immune stimulation. Both aggregates showed enhanced aggregation and encapsulation of c-di-GMP coupled with effective cellular uptake and response, demonstrating the promise of these formulations in therapeutic contexts. Further research should focus on optimizing and determining the release kinetics of c-di-GMP, exploring molecular interactions that drive encapsulation and aggregation, comparing amyloid treatments against commonly used lipid nanoparticles for nucleotide delivery, and evaluating these immunostimulatory effects in more complex biological models. The results found in our paper suggest that amyloid-based depots may improve the efficacy of c-di-GMP delivery for immunostimulatory therapies, offering a promising alternative to conventional methods.

## Data Availability

The data relevant to the results of the article will be made available by the authors, without any restrictions.

## References

[1] Romling U., Amikam D. (2006). Cyclic di-GMP as a second messenger. Curr. Opin. Microbiol..

[2] Nakamura T., Miyabe H., Hyodo M., Sato Y., Hayakawa Y., Harashima H. (2015). Liposomes loaded with a STING pathway ligand, cyclic di-GMP, enhance cancer immunotherapy against metastatic melanoma. J. Control Release.

[3] Karaolis D.K., Means T.K., Yang D., Takahashi M., Yoshimura T., Muraille E., Philpott D., Schroeder J.T., Hyodo M., Hayakawa Y. (2007). Bacterial c-di-GMP is an immunostimulatory molecule. J. Immunol..

[4] Woo S.R., Fuertes M.B., Corrales L., Spranger S., Furdyna M.J., Leung M.Y., Duggan R., Wang Y., Barber G.N., Fitzgerald K.A. (2014). STING-dependent cytosolic DNA sensing mediates innate immune recognition of immunogenic tumors. Immunity.

[5] Burdette D.L., Monroe K.M., Sotelo-Troha K., Iwig J.S., Eckert B., Hyodo M., Hayakawa Y., Vance R.E. (2011). STING is a direct innate immune sensor of cyclic di-GMP. Nature.

[6] Shu C., Yi G., Watts T., Kao C.C., Li P. (2012). Structure of STING bound to cyclic di-GMP reveals the mechanism of cyclic dinucleotide recognition by the immune system. Nat. Struct. Mol. Biol..

[7] Wang H.H., Tsourkas A. (2019). Cytosolic delivery of inhibitory antibodies with cationic lipids. Proc. Natl. Acad. Sci. USA.

[8] Zhang Z., Liu J., Xiao M., Zhang Q., Liu Z., Liu M., Zhang P., Zeng Y. (2023). Peptide nanotube loaded with a STING agonist, c-di-GMP, enhance cancer immunotherapy against melanoma. Nano Res..

[9] Chen Y.P., Xu L., Tang T.W., Chen C.H., Zheng Q.H., Liu T.P., Mou C.Y., Wu C.H., Wu S.H. (2020). STING Activator c-di-GMP-Loaded Mesoporous Silica Nanoparticles Enhance Immunotherapy Against Breast Cancer. ACS Appl. Mater. Interfaces.

[10] Abdelrahman S., Alghrably M., Lachowicz J.I., Emwas A.H., Hauser C.A.E., Jaremko M. (2020). “What Doesn’t Kill You Makes You Stronger”: Future Applications of Amyloid Aggregates in Biomedicine. Molecules.

[11] Li J., McQuade T., Siemer A.B., Napetschnig J., Moriwaki K., Hsiao Y.S., Damko E., Moquin D., Walz T., McDermott A. (2012). The RIP1/RIP3 necrosome forms a functional amyloid signaling complex required for programmed necrosis. Cell.

[12] Chen T., Wang Y., Xie J., Qu X., Liu C. (2022). Lysozyme Amyloid Fibril-Integrated PEG Injectable Hydrogel Adhesive with Improved Antiswelling and Antibacterial Capabilities. Biomacromolecules.

[13] Iconomidou V.A., Hamodrakas S.J. (2008). Natural protective amyloids. Curr. Protein Pept. Sci..

[14] Waku T., Tanaka N. (2017). Recent advances in nanofibrous assemblies based on β-sheet-forming peptides for biomedical applications. Polym. Int..

[15] Lai Y.R., Wang S.S., Hsu T.L., Chou S.H., How S.C., Lin T.H. (2023). Application of Amyloid-Based Hybrid Membranes in Drug Delivery. Polymers.

[16] Diaz-Caballero M., Fernandez M.R., Navarro S., Ventura S. (2018). Prion-based nanomaterials and their emerging applications. Prion.

[17] Pena-Diaz S., Olsen W.P., Wang H., Otzen D.E. (2024). Functional Amyloids: The Biomaterials of Tomorrow?. Adv. Mater..

[18] Torok M. (2022). Amyloids in synthetic applications. Sci. Rep..

[19] Yang L., Li H., Yao L., Yu Y., Ma G. (2019). Amyloid-Based Injectable Hydrogel Derived from Hydrolyzed Hen Egg White Lysozyme. ACS Omega.

[20] Swaminathan R., Ravi V.K., Kumar S., Kumar M.V., Chandra N. (2011). Lysozyme: A model protein for amyloid research. Adv. Protein Chem. Struct. Biol..

[21] Nawaz N., Wen S., Wang F., Nawaz S., Raza J., Iftikhar M., Usman M. (2022). Lysozyme and Its Application as Antibacterial Agent in Food Industry. Molecules.

[22] Derde M., Lechevalier V., Guerin-Dubiard C., Cochet M.F., Jan S., Baron F., Gautier M., Vie V., Nau F. (2013). Hen egg white lysozyme permeabilizes Escherichia coli outer and inner membranes. J. Agric. Food Chem..

[23] Morimoto R., Horita M., Yamaguchi D., Nakai H., Nakano S.I. (2022). Evaluation of weak interactions of proteins and organic cations with DNA duplex structures. Biophys. J..

[24] Lv M., Wang M., Lu K., Peng L., Zhao Y. (2019). DNA/Lysozyme-binding affinity study of novel peptides from TAT (47–57) and BRCA1 (782–786) in vitro by spectroscopic analysis. Spectrochim. Acta A Mol. Biomol. Spectrosc..

[25] Steinrauf L.K., Shiuan D., Yang W.J., Chiang M.Y. (1999). Lysozyme association with nucleic acids. Biochem. Biophys. Res. Commun..

[26] Ragland S.A., Criss A.K. (2017). From bacterial killing to immune modulation: Recent insights into the functions of lysozyme. PLoS Pathog..

[27] Ismail M., Kanapathipillai M. (2024). Novel Ultrasound-Responsive Amyloid Formulation. Pharmaceuticals.

[28] Wu X.L., Hu H., Dong X.Q., Zhang J., Wang J., Schwieters C.D., Liu J., Wu G.X., Li B., Lin J.Y. (2021). The amyloid structure of mouse RIPK3 (receptor interacting protein kinase 3) in cell necroptosis. Nat. Commun..

[29] Hu H., Wu X., Wu G., Nan N., Zhang J., Zhu X., Zhang Y., Shu Z., Liu J., Liu X. (2021). RIP3-mediated necroptosis is regulated by inter-filament assembly of RIP homotypic interaction motif. Cell Death Differ..

[30] Wu X., Ma Y., Zhao K., Zhang J., Sun Y., Li Y., Dong X., Hu H., Liu J., Wang J. (2021). The structure of a minimum amyloid fibril core formed by necroptosis-mediating RHIM of human RIPK3. Proc. Natl. Acad. Sci. USA.

[31] Alamgir A., Ghosal S., DeLisa M.P., Alabi C.A. (2024). Bioreversible Anionic Cloaking Enables Intracellular Protein Delivery with Ionizable Lipid Nanoparticles. ACS Cent. Sci..

[32] Zuris J.A., Thompson D.B., Shu Y., Guilinger J.P., Bessen J.L., Hu J.H., Maeder M.L., Joung J.K., Chen Z.Y., Liu D.R. (2015). Cationic lipid-mediated delivery of proteins enables efficient protein-based genome editing in vitro and in vivo. Nat. Biotechnol..

[33] Kulkarni J.A., Witzigmann D., Chen S., Cullis P.R., van der Meel R. (2019). Lipid Nanoparticle Technology for Clinical Translation of siRNA Therapeutics. Acc. Chem. Res..

[34] Frare E., Mossuto M.F., de Laureto P.P., Tolin S., Menzer L., Dumoulin M., Dobson C.M., Fontana A. (2009). Characterization of oligomeric species on the aggregation pathway of human lysozyme. J. Mol. Biol..

[35] Sooreshjani M.A., Gursoy U.K., Aryal U.K., Sintim H.O. (2018). Proteomic analysis of RAW macrophages treated with cGAMP or c-di-GMP reveals differentially activated cellular pathways. RSC Adv..

[36] Yildiz S., Alpdundar E., Gungor B., Kahraman T., Bayyurt B., Gursel I., Gursel M. (2015). Enhanced immunostimulatory activity of cyclic dinucleotides on mouse cells when complexed with a cell-penetrating peptide or combined with CpG. Eur. J. Immunol..

[37] Gauley J., Pisetsky D.S. (2010). The release of microparticles by RAW 264.7 macrophage cells stimulated with TLR ligands. J. Leukoc. Biol..

[38] Yan W., Yan Y., Luo X., Dong Y., Liang G., Miao H., Huang Z., Jiang H. (2024). Lipopolysaccharide (LPS)-induced inflammation in RAW264.7 cells is inhibited by microRNA-494-3p via targeting lipoprotein-associated phospholipase A2. Eur. J. Trauma. Emerg. Surg..

[39] Sawaya M.R., Hughes M.P., Rodriguez J.A., Riek R., Eisenberg D.S. (2021). The expanding amyloid family: Structure, stability, function, and pathogenesis. Cell.

[40] Westermark P., Benson M.D., Buxbaum J.N., Cohen A.S., Frangione B., Ikeda S., Masters C.L., Merlini G., Saraiva M.J., Sipe J.D. (2007). A primer of amyloid nomenclature. Amyloid.

[41] Yakupova E.I., Bobyleva L.G., Shumeyko S.A., Vikhlyantsev I.M., Bobylev A.G. (2021). Amyloids: The History of Toxicity and Functionality. Biology.

[42] Kushnirov V.V., Vishnevskaya A.B., Alexandrov I.M., Ter-Avanesyan M.D. (2007). Prion and nonprion amyloids: A comparison inspired by the yeast Sup35 protein. Prion.

[43] Si K., Lindquist S., Kandel E.R. (2003). A neuronal isoform of the aplysia CPEB has prion-like properties. Cell.

[44] Guyonnet B., Egge N., Cornwall G.A. (2014). Functional amyloids in the mouse sperm acrosome. Mol. Cell Biol..

[45] Fowler D.M., Koulov A.V., Alory-Jost C., Marks M.S., Balch W.E., Kelly J.W. (2006). Functional amyloid formation within mammalian tissue. PLoS Biol..

[46] Cao C., Tian L., Li J., Raveendran R., Stenzel M.H. (2024). Mix and Shake: A Mild Way to Drug-Loaded Lysozyme Nanoparticles. ACS Appl. Mater. Interfaces.

